# Actual timing versus GPs’ perceptions of optimal timing of advance care planning: a mixed-methods health record-based study

**DOI:** 10.1186/s12875-022-01940-3

**Published:** 2022-12-13

**Authors:** Willemijn Tros, Jenny T. van der Steen, Janine Liefers, Reinier Akkermans, Henk Schers, Mattijs E. Numans, Petra G. van Peet, A. Stef Groenewoud

**Affiliations:** 1 Department of Public Health and Primary Care, Leiden University Medical Center (LUMC), Leiden, the Netherlands; 2 Department of Primary and Community Care, Radboud university medical center, Nijmegen, the Netherlands; 3 Radboud Institute for Health Sciences, Scientific Center for Quality of Healthcare, Radboud university medical center, Nijmegen, the Netherlands

**Keywords:** Advance care planning, Cancer, Organ failure, Multimorbidity, General practice, Electronic health record

## Abstract

**Background:**

Timely initiation of advance care planning (ACP) in general practice is challenging, especially in patients with non-malignant conditions. Our aim was to investigate how perceived optimal timing of ACP initiation and its triggers relate to recorded actual timing in patients with cancer, organ failure, or multimorbidity.

**Methods:**

In this mixed-methods study in the Netherlands, we analysed health records selected from a database with primary care routine data and with a recorded ACP conversation in the last two years before death of patients who died with cancer, organ failure, or multimorbidity. We compared actual timing of ACP initiation as recorded in health records of 51 patients with the perceived optimal timing as determined by 83 independent GPs who studied these records. Further, to identify and compare triggers for GPs to initiate ACP, we analysed the health record documentation around the moments of the recorded actual timing of ACP initiation and the perceived optimal timing of ACP initiation. We combined quantitative descriptive statistics with qualitative content analysis.

**Results:**

The recorded actual timing of ACP initiation was significantly closer to death than the perceived optimal timing in patients with cancer (median 88 vs. 111 days before death (p = 0.049)), organ failure (227 vs. 306 days before death (*p* = 0.02)) and multimorbidity (113 vs. 338 days before death (*p* = 0.006)). Triggers for recorded actual versus perceived optimal timing were similar across the three groups, the most frequent being ‘expressions of patients’ reflections or wishes’ (14% and 14% respectively) and ‘appropriate setting’ (10% and 13% respectively).

**Conclusion:**

ACP in general practice was initiated and recorded later in the illness trajectory than considered optimal, especially in patients with organ failure or multimorbidity. As triggers were similar for recorded actual and perceived optimal timing, we recommend that GPs initiate ACP shortly after a trigger is noticed the first time, rather than wait for additional or more evident triggers when the illness is in an advanced stage.

**Supplementary Information:**

The online version contains supplementary material available at 10.1186/s12875-022-01940-3.

## Background

The importance of patient centeredness of care at the end of life is increasingly acknowledged. Patients, bereaved family, and healthcare professionals all express that it is essential for a good end of life that patients’ wishes are met and a sense of control is achieved [1–3]. Advance care planning (ACP) is believed to play a role in improving the quality of end-of-life care [4–6]. ACP is a process that enables patients to specify and share their values, goals and preferences for future medical treatment and care [7, 8]. This process is perceived as a task that is typically suited to general practitioners (GPs) as they usually have a longstanding relationship with patients enlisted in their practice [9, 10]. However, there is a wide variety and generally low uptake of ACP and it mainly takes place reactively and thus late in the disease trajectory of patients, particularly in patients with non-malignant conditions such as organ failure, dementia or multimorbidity [11–15].

Inconsistent application of ACP in general practice may relate to timing difficulties. In non-malignant diseases, such as heart failure, key moments to initiate ACP and the life-limiting nature of the diseases are not always apparent to GPs [9, 16, 17]. Furthermore, GPs reported concerns about taking away hope from patients [9]. Yet, timely initiation of ACP may prevent ad-hoc end-of-life treatment that tends not to reflect patients´ wishes [18].

Recent research has studied either the recorded actual timing of ACP initiation in daily practice [19], or GPs’ perceptions of optimal timing of ACP initiation [20]. Understanding matches and mismatches between the two may, however, inform better timing of ACP conversations in practice. The aim of the current study is to investigate how perceived optimal timing of ACP initiation and its triggers relate to recorded actual timing for patients with cancer, organ failure, or multimorbidity in general practice.

## Methods

### Study design and setting

In this mixed-method health record review study, we used records of patients who died with cancer, organ failure or multimorbidity [19, 20]. We compared the actual timing of ACP initiation in general practice, as recorded in the patients’ health records, with the perceived optimal timing according to independent GPs who examined the last two years of the health records retrospectively. Further, to identify and compare triggers for GPs to initiate ACP, we analysed the health record documentation around the moments of the recorded actual timing of ACP initiation and the perceived optimal timing of ACP initiation. The actual and the perceived optimal timing of ACP initiation were determined in previously published after death health record studies [19, 20].

### Data source

Pseudonymized patient health records were selected from FaMe-net, a database with primary care routine data collected in the region of Nijmegen, the Netherlands, covering patient health record data from seven general practices. We selected records of patients who died between 2003 and 2016 that contained patient characteristics, GP reports, correspondence to and from other healthcare providers, laboratory values, and medication prescriptions. We used data from the last two years of life and excluded health records of patients with fewer than 2 years documented before death. Further, records of patients under the age 18 and those with a diagnosis of dementia were excluded. In 150 randomly sampled health records equally distributed across the seven general practices, we identified three patient groups based on different illness trajectories [21, 22]: (i) patients who died with cancer, whose decline is generally evident and progressive. (ii) patients who died with organ failure (heart failure, COPD, kidney failure, liver failure and chronic-progressive neurological illness such as Parkinson’s or ALS), whose decline is characterized by long-term limitations with intermittent worsening of symptoms and some recovery, often with a rather sudden death. (iii) older patients (age > 65) who died with multiple (> 2) chronic diseases, other than cancer and organ failure (i.e., multimorbidity), whose decline is generally prolonged and gradual. Patients who could be allocated to more than one group, were allocated to the first fitting group in the following order: cancer (1), organ failure (2), multimorbidity (3). Allocation was based on verified diagnosis from recorded medical history. We excluded records that could not be assigned to any of these three groups, as death may have been a ‘sudden death’ (unpredictable, acute illness or trauma) and records with unknown cause of death.

### Data on recorded actual timing of ACP initiation

In a previous study, the authors (among them SG and HS) examined the documentation of ACP for patients with cancer, organ failure and multimorbidity [19]. In this study the same selection procedure as described above was followed and resulted in 119 included health records. Data abstracted from the health records included the presence of ACP and the timing of the first ACP conversation. ACP is referred to, consistent with a recent international consensus definition, as proactive conversations, registrations or actions such as conversations on treatment preferences for future care and conversations regarding prognosis, personal wishes and goals or concerns, and hopes for the future [8].

### Data on perceived optimal timing of ACP initiation

Another study we use data from in the current research, aimed to identify the optimal moment, and reasons to initiate ACP in patients with cancer, organ failure and multimorbidity. In this study, a selection of 90 health records (30 records per patient group), selected with the same process as above, was assessed by 83 independent GPs. GPs were recruited from networks with and without a particular interest in end-of-life care from various geographical areas and through snowballing, to ensure diversity in GPs’ backgrounds and took place between October 31^st^, 2020 and January 10^th^, 2021.The included GPs, with an average of 15 year experience as GP and with 24% of them having an additional expertise in palliative care, assessed optimal timing of ACP initiation The researchers first removed all indicators of actual performed ACP in these records. Then, the GPs reviewed, independently of each other, three patient health records (one from each patient group) in an online environment to determine what time they perceived as the optimal time to initiate ACP through thorough assessment of all documentation (patient characteristics, GP reports, correspondence to and from other healthcare providers, laboratory values, and medication prescriptions) in the last two years of life. Furthermore, they were asked to explain why they thought this would be the optimal time to initiate ACP [20].

### Data analysis

**Fig. 1:**
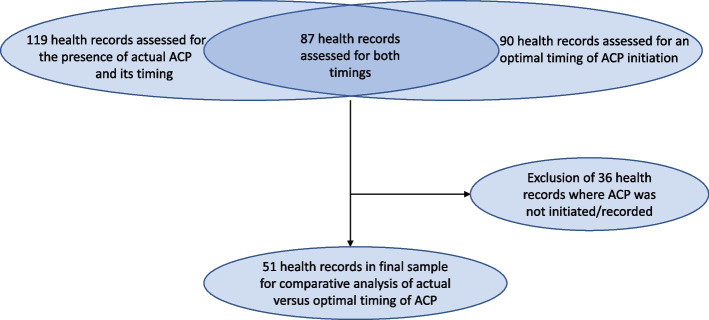
Selection of health records for comparative analysis

In the current study, we selected and analysed health records that were included in both previous studies. We excluded health records where ACP was not recorded (Fig. 1). We compared the recorded actual timing of ACP initiation with the perceived optimal timing of ACP initiation, as identified in previous performed studies [19, 20]. The perceived optimal timing of ACP initiation for each health record was determined by taking the average of the optimal timing as identified by the maximum of three independent GPs who assessed that same record. We present recorded actual timing of ACP initiation and optimal timing in the three patient groups (cancer, organ failure, and multimorbidity) referring to the median number of days and interquartile range (IQR) between timing and patients’ death. Differences between actual and optimal timing of ACP initiation were tested for every patient group using the Wilcoxon Signed Rank test to accommodate skewed distributions. A p-value of < 0.05, based on two-sided tests was considered statistically significant. We performed quantitative analysis in SPSS version 25 (IBM, 2017).

To identify what seems to have triggered the GPs to initiate ACP, we conducted directed qualitative content analysis [23], with the codes and categories developed in the previous study [20] on health record documentation around the recorded actual timing and the perceived optimal timing of ACP initiation. Texts were abstracted to Microsoft Excel 2016 for coding. One researcher (WT) first coded the texts around the perceived optimal timing of ACP and then the texts around the first recorded ACP conversation. Ambiguous cases (equal to 20%) were discussed extensively with a second researcher (PvP), whereafter codes in all records were adapted as needed. Subsequently, codes and themes were counted for and compared between the actual and the optimal timing of ACP initiation, within and between the three groups.

## Results

The characteristics of the 51 patients whose health record were analysed are shown in Table 1.

**Table 1:** Characteristics of the patients whose health records were analysed

	**Total** **(n = 51)**	**Cancer** **(n = 24)**	**Organ failure** **(n = 16)**	**Multimorbidity** **(n = 11)**
**Female sex:** *% (n)*	61 (31)	63 (15)	56 (9)	64 (7)
**Age in years at time of death:** *median (IQR)*	82 (12)	75 (17)	85 (9)	89 (16)

### Recorded actual vs. perceived optimal timing of ACP initiation

**Fig. 2:**
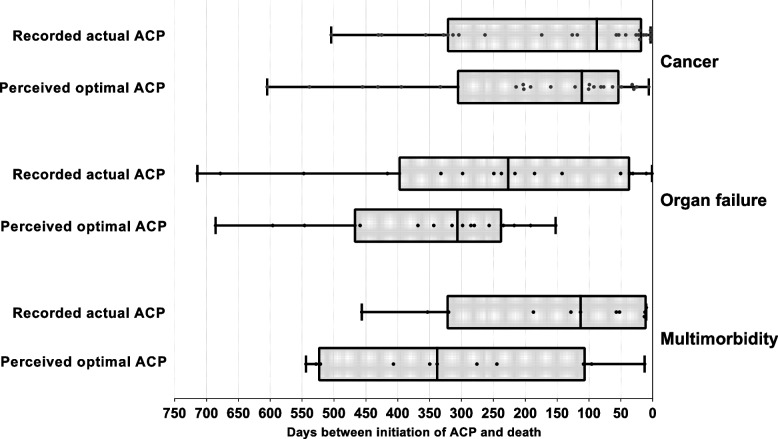
Distribution plot of recorded actual ACP timing in general practice vs. perceived optimal ACP timing as determined by independent GPs in cancer (n = 24), organ failure (n = 16) and multimorbidity (n = 11). Each point in the plot of perceived optimal ACP represents a health record assessed by a maximum of three independent GPs: in the cancer group, 18 health records were reviewed by three GPs and 6 by two GPs; in the organ failure group, 12 records were reviewed by three GPs, two records by two GPs and two records by one GP; in the multimorbidity group, 9 records were reviewed by three GPs, one record by two GP and one record by one GP

The median recorded actual timing of ACP initiation was 128 days before death (IQR 299). This was significantly closer to death than the perceived optimal timing (median 244 days before death, IQR 307; *p* < 0.001). In all patient groups, the recorded actual timing of ACP initiation was significantly closer to death than the perceived optimal timing (cancer, median (IQR): 88 (299) vs. 111 (248), *p* = 0.049; organ failure, median (IQR): 227 (356) vs. 306 (225), *p* = 0.020; multimorbidity, median (IQR): 113 (307) vs. 338 (413), *p* = 0.006; Fig. 2).

### Qualitative analysis of the moment of the actual vs. perceived optimal ACP initiation

**Fig. 3:**
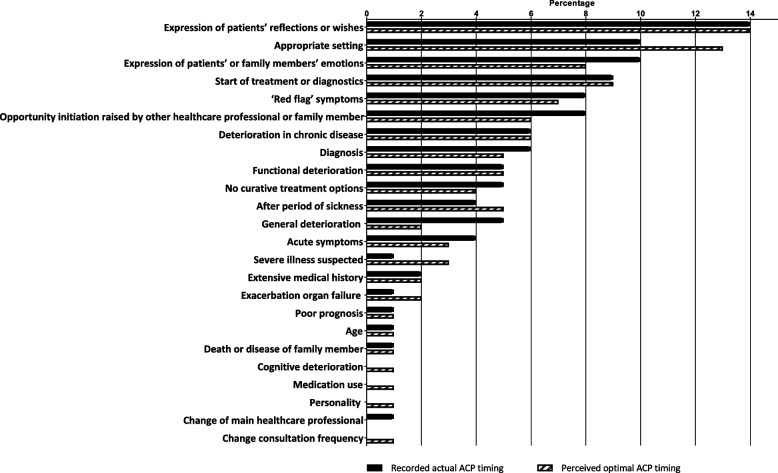
Triggers around the moments of the recorded actual and the perceived optimal ACP initiation. ‘Appropriate setting’ constitutes a period of relative wellness, a setting with adequate time, or presence of a family member

Our qualitative analysis of moments of recorded actual versus perceived optimal ACP conversations across all 51 health records showed that actual ACP was most frequently initiated when patients expressed their reflections or wishes (14%), when the setting was appropriate (10%; e.g., period of relative wellness, a setting with adequate time, or presence of a family member), and when patients or family members expressed their emotions (10%) (Fig. 3). Perceived optimal timing of ACP initiation was most frequently identified when patients expressed their reflections or wishes (14%), when the setting was appropriate (13%), and when treatment or diagnostics were started (9%). Across all health records, GPs slightly more often reported an appropriate setting for the optimal timing of ACP initiation compared to the actual timing for ACP initiation (13% vs. 10%). Further, actual ACP was more frequently initiated as a response to ‘general deterioration’ compared to perceived optimal timing of ACP (5% vs. 2%) (see Additional file 1 for the complete results of the qualitative analysis).

The distribution of triggers for ACP (what possibly made GPs decide to initiate ACP) per patient group were largely similar for recorded actual and perceived optimal timing of ACP initiation (Table 2). In patients who died with cancer, actual and perceived optimal timing of ACP initiation was most frequently triggered by a specific moment ‘in the timeline of the disease’ (e.g., diagnosis, no curative treatment options available, or at start of treatment or diagnostics), which was less often a trigger for actual than for perceived optimal timing (32% vs. 39%). ‘Symptoms indicating deterioration’ were more often a trigger in actual timing for ACP initiation than in perceived optimal timing (25% vs. 17%). There were also modest differences in the other patient groups: for example, in health records of patients who died with organ failure, ‘symptoms indicating deterioration’ were less often a trigger for actual timing of ACP initiation (24%) than for perceived optimal timing (29%), while the reverse applied to the multimorbidity group (44% actual vs. 36% optimal timing).

**Table 2:** Triggers (main categories) around the moments of the recorded actual and the perceived optimal ACP timing

	Total (n = 51)			Cancer (n = 24)			Organ failure (n = 16)			Multimorbidity (n = 11)		
*Timing (number of triggers identified)* Triggers, % of total n identified triggers	*Recorded actual* *(n* = *144)*	*Perceived optimal (n* = 388*)		*Recorded actual (n* = *71)*	*Perceived optimal (n* = 197*)		*Recorded actual (n* = *41)*	*Perceived optimal (n* = 114*)		*Recorded actual (n* = *32)*	*Perceived optimal (n* = 77*)	
	%	%		%	%		%	%		%	%	
In timeline of the disease^a^	25	28		32	39		20	23		16	9	
Symptoms indicating deterioration^b^	29	24		25	17		24	29		44	36	
Mental and spiritual health aspects^c^	24	23		25	24		22	22		25	23	
Patient characteristics^d^	3	3		0	2		10	4		0	7	
Appropriate setting^**^	10	13		10	13		10	12		9	14	
Social context^e^	1	2		1	1		2	3		0	4	
Opportunity for initiation raised by other healthcare professional or family member	8	6		10	6		12	7		6	7	

^*^as perceived optimal timing of ACP initiation is identified by up to 3 GPs per health record, the total number of identified triggers is high, compared to total number of triggers of actual timing

^**^ constitutes period of relative wellness, a setting with adequate time, or presence of a family member

^a ^includes triggers ‘Start of treatment or diagnostics’, ‘Diagnosis’, ‘After period of sickness’, ‘No curative treatment options’, ‘Suspicion of severe illness’, ‘Poor prognosis’

^b ^includes triggers ‘Deterioration in chronic disease’, ‘‘Red flag’ symptoms’, ‘Functional deterioration’, ‘Acute symptoms’, ‘General deterioration’, ‘Exacerbation organ failure ‘, ‘Cognitive deterioration’, ‘Change in need for consultation’

^c^ includes triggers ‘Expression of patients’ reflections or wishes’, ‘Expression of patients’ or family members’ emotions’, ‘Intrinsic personality and care avoidance’

^d^ includes triggers ‘Extensive medical history’, ‘Age’, ‘Medication use’

^e^ includes triggers ‘Death or disease of family member’, ‘Social vulnerability’, ‘Change of main healthcare professional’

## Discussion

We compared the recorded actual timing of ACP initiation in general practice in patients who died with cancer, organ failure, or multimorbidity, with the perceived optimal timing of ACP initiation as assessed by peer GPs. When initiated, ACP in general practice, especially in patients with organ failure or multimorbidity, was initiated significantly later than what was considered optimal by their independent peers. However, across the three patient groups, the triggers for recorded actual timing and for perceived optimal timing of ACP initiation were quite similar. Nevertheless, ‘appropriate setting’ (e.g., period of relative wellness, a setting with adequate time, or presence of a family member) was slightly more often a trigger for perceived optimal timing of ACP initiation than for initiating ACP in actual practice. Further, optimal timing of ACP was less often in response to ‘general deterioration’ compared to actual ACP.

### Interpretation of results in the light of existing literature

Our findings are in line with previous studies indicating that GPs often initiate ACP late, when the disease has reached a critical stage [9, 13, 16, 24]. Reasons given for suboptimal timing are that proactively initiating ACP can be difficult as patients are not always open to having them and healthcare providers are afraid to take away a patients’ hope for a cure [9].

Nevertheless, the fear of causing anxiety and catching patients off guard and thereby damaging the patient-doctor relationship might not be warranted. Previous research found that the majority of the oncology patients preferred to have ACP discussions early, before their prognosis worsened. Additionally, almost half of the patients wished these conversations had taken place even before they were diagnosed with cancer [25]. In another study, participants who received ACP shortly after their diagnosis of advanced cancer, acknowledged that the conversation was emotional, but not burdensome and felt it helped them [26]. Studies conducted in patients with heart failure and healthy older people, although few, suggest that patients might be more open to ACP than physicians think [27, 28].

It has also been shown that patients do not have to be ready for all ACP topics to be able to participate in ACP conversations [29]. Additionally, readiness can alternate during the course of the ACP conversation. Thus, it is important to verify who wants what information at that specific moment and tailor the conversation to the patients’ needs, and to not postpone initiating ACP until patients are ready for all ACP topics [30].

To ensure triggers to initiate ACP are not missed, screening tools such as SPICT, RADPAC or the ‘surprise question’ can be used [31–33]. In these tools some triggers are also used such as frequent hospital admissions, lower functional status and weight loss. However these tools aim to identify patients who are in need for palliative care because they are at high risk of dying, which is different from identifying patients who could benefit from ACP. We found additional triggers to identify patients who are in need of ACP such as patients expressing wishes themselves, other health care professionals raises opportunity to initiate ACP or the opportunity of a setting with adequate time.

### Strengths and limitations

To the best of our knowledge, this is the first study to research how perceived optimal timing of ACP initiation and its triggers relates to recorded actual timing in general practice. Our original, mixed-method approach indicates a difference between actual timing of ACP initiation in general practice and the optimal ACP timing as indicated by independent GPs. The quantitative findings were enhanced with qualitative findings showing that that the nature and distribution of the many possible triggers initiating ACP in practice did not differ much from the triggers related to the optimal moment. Also, some limitations must be acknowledged. Actual ACP timing in our analysis was defined as the first recorded ACP conversation. It is possible that ACP was conducted earlier without recording it. However, we expect that GPs affiliated with a practice-based research network have a better registration routine than other GPs. Furthermore, in order to minimalize the risk of missing the initiation of ACP the full health record was assessed including free text fields.

Another limitation is that optimal timing involves a subjective judgement. However, by evaluation of multiple GPs with variable experience and expertise, on the same health records, we could capture an inter-subjective understanding of the optimal timing of ACP initiation. Further, the follow-back perspective removed prognostic insecurity (as the GPs knew when the patient actually died) and therefore may have reduced variability in the identification of the optimal timing. Additionally, we could not include many patients per group, perhaps due to low prevalence of ACP in general practice, as previously described in literature [13–15] This underlines the importance of our research even more.

Last, the data of recorded actual timing of ACP initiation comprises real life data between 2003 and 2016, whereas perceived optimal timing of ACP initiation was determined by GPs more recently, in 2021. ACP practice and how it is being perceived might have changed in this period, which may also partly explain the differences between perceived optimal timing and recorded actual timing of ACP initiation [8, 34].

### Implications for practice and future research

Our results suggest that in most cases, triggers to initiate ACP are already in place before ACP is initiated in general practice. It is important to act upon triggers at an early stage adopting a proactive approach, and not postpone initiating ACP or wait for additional triggers.

As triggers to initiate ACP can be subtle and are sometimes either missed by GPs or not translated into action in real life situations, future research should focus on developing practical tools that automatically detect these triggers in electronic health records and support GPs in deciding when to initiate ACP. Artificial Intelligence, algorithms and flagging aids could be useful.[35] Whether such practical tools will eventually lead to more and more timely initiation of ACP in general practice, especially in patients with non-malignant diseases, should be investigated further. In addition, more research is needed that solicits patients’ views on ACP timing.

## Conclusions

This study shows that ACP in general practice was initiated and recorded later in the illness trajectory than considered optimal, especially in patients with organ failure and patients with multimorbidity. The triggers for recorded actual timing of ACP and the perceived optimal timing appeared to be similar. Because the timely initiation of ACP could optimize care at the end of life, we recommend that GPs initiate ACP when a trigger first becomes apparent rather than wait for additional or more evident triggers, or at least consider inviting the patient to an ACP conversation. It is hoped that this will promote a more proactive approach in initiating ACP in particular for patients with non-malignant diseases.

## Supplementary Information

**Additional file 1.** 

## Data Availability

Data are available upon request from the corresponding author on reasonable request, except the content of the patient health records from FameNet as this data is not publicly available.

## Abbreviation

ACP: Advance Care Planning; GP: General practitioner
