# Supplementary material for: Actual timing versus GPs’ perceptions of optimal timing of advance care planning: a mixed-methods health record-based study
Source: BMC Prim Care. 2022 Dec 13;23:321. doi: 10.1186/s12875-022-01940-3 (PMC9749277; doi:10.1186/s12875-022-01940-3)
Supplement: Supplementary file 1 — Additional file 1. [file 12875_2022_1940_MOESM1_ESM.docx]

**Additional file 1**

Supplementary table 1. Triggers around the moments of the actual vs. perceived optimal ACP initiation per patient group

|  | Total (n=51) | | | |  | Cancer (n=24) | | |  | | Organ failure (n=16) | | | |  | Multimorbidity (n=11) | | | | |  | | |
| --- | --- | --- | --- | --- | --- | --- | --- | --- | --- | --- | --- | --- | --- | --- | --- | --- | --- | --- | --- | --- | --- | --- | --- |
| *Timing (number of triggers identified)*  Triggers, % of total n identified triggers | *Recorded actual (n=144)* | | *Perceived optimal (n=388*)* | | | *Recorded actual (n=71)* | | *Perceived optimal (n=197*)* | | | *Recorded actual (n=41)* | | *Perceived optimal (n=114*)* | | | *Recorded actual (n=32)* | | *Perceived optimal (n=77*)* | | | |  |  |
|  | % | n | % | n | | % | n | % | | n | % | n | % | n | | % | n | | % | n | | |  |
| In timeline of the disease: | **25** | 36 | **28** | 109 | | **32** | 23 | **39** | | 76 | **20** | 8 | **23** | 26 | | **16** | 5 | | **9** | **7** | | |  |
| Start of treatment or diagnostics | 9 | 13 | 9 | 35 | | 11 | 8 | 14 | | 27 | 5 | 2 | 5 | 6 | | 9 | 3 | | 3 | 2 | | |  |
| Diagnosis | 6 | 8 | 5 | 21 | | 9 | 6 | 9 | | 17 | 2 | 1 | 4 | 4 | | 3 | 1 | | 0 | 0 | | |  |
| After period of sickness | 4 | 5 | 5 | 20 | | 1 | 1 | 4 | | 7 | 7 | 3 | 11 | 12 | | 3 | 1 | | 1 | 1 | | |  |
| No curative treatment options | 5 | 7 | 4 | 17 | | 9 | 6 | 8 | | 16 | 2 | 1 | 0 | 0 | | 0 | 0 | | 1 | 1 | | |  |
| *S*uspicion of severe illness | 1 | 2 | 3 | 13 | | 1 | 1 | 4 | | 7 | 2 | 1 | 3 | 3 | | 0 | 0 | | 4 | 3 | | |  |
| Poor prognosis | 1 | 1 | 1 | 3 | | 1 | 1 | 1 | | 2 | 0 | 0 | 1 | 1 | | 0 | 0 | | 0 | 0 | | |  |
| Symptoms indicating deterioration: | **29** | 42 | **24** | 92 | | **25** | 18 | **17** | | 33 | **24** | 10 | **29** | 33 | | **44** | 14 | | **36** | 28 | | |  |
| Deterioration in chronic disease | 6 | 9 | 6 | 23 | | 9 | 6 | 8 | | 15 | 5 | 2 | 5 | 6 | | 3 | 1 | | 3 | 2 | | |  |
| ‘Red flag’ symptoms | 8 | 11 | 7 | 20 | | 10 | 7 | 5 | | 9 | 2 | 1 | 3 | 3 | | 9 | 3 | | 10 | 8 | | |  |
| Functional deterioration | 5 | 7 | 5 | 19 | | 3 | 2 | 2 | | 3 | 2 | 1 | 8 | 9 | | 13 | 4 | | 9 | 7 | | |  |
| Acute symptoms | 4 | 6 | 3 | 12 | | 1 | 1 | 1 | | 1 | 10 | 4 | 5 | 6 | | 3 | 1 | | 7 | 5 | | |  |
| General deterioration | **5** | **7** | **2** | **8** | | 3 | 2 | 2 | | 3 | 0 | 0 | 1 | 1 | | 16 | 5 | | 5 | 4 | | |  |
| Exacerbation organ failure | 1 | 2 | 2 | 6 | | 0 | 0 | 1 | | 1 | 5 | 2 | 4 | 5 | | 0 | 0 | | 0 | 0 | | |  |
| Cognitive deterioration | 0 | 0 | 1 | 3 | | 0 | 0 | 0 | | 0 | 0 | 0 | 2 | 2 | | 0 | 0 | | 1 | 1 | | |  |
| Change in need for consultation | 0 | 0 | 1 | 3 | | 0 | 0 | 1 | | 1 | 0 | 0 | 1 | 1 | | 0 | 0 | | 1 | 1 | | |  |
| Mental and spiritual health aspects: | **24** | 35 | **23** | 90 | | **25** | 18 | **24** | | 47 | **22** | 9 | **22** | 25 | | **25** | 8 | | **23** | 18 | | |  |
| Expression of patients’ reflections or wishes | 14 | 20 | 14 | 56 | | 17 | 12 | 15 | | 29 | 12 | 5 | 13 | 15 | | 9 | 3 | | 16 | 12 | | |  |
| Expression of patients’ or family members’ emotions | 10 | 15 | 8 | 31 | | 9 | 6 | 9 | | 18 | 10 | 4 | 6 | 7 | | 16 | 5 | | 8 | 6 | | |  |
| Intrinsic personality and care avoidance | 0 | 0 | 1 | 3 | | 0 | 0 | 0 | | 0 | 0 | 0 | 3 | 3 | | 0 | 0 | | 0 | 0 | | |  |
| Patient characteristics: | **3** | 4 | **3** | 13 | | **0** | 0 | **2** | | 3 | **10** | 4 | **4** | 5 | | **0** | 0 | | **7** | 5 | | |  |
| Extensive medical history | 2 | 3 | 2 | 7 | | 0 | 0 | 1 | | 1 | 7 | 3 | 4 | 5 | | 0 | 0 | | 1 | 1 | | |  |
| Age | 1 | 1 | 1 | 3 | | 0 | 0 | 0 | | 0 | 2 | 1 | 0 | 0 | | 0 | 0 | | 4 | 3 | | |  |
| Medication use | 0 | 0 | 1 | 3 | | 0 | 0 | 1 | | 2 | 0 | 0 | 0 | 0 | | 0 | 0 | | 1 | 1 | | |  |
| Appropriate setting: | **10** | 14 | **13** | 50 | | **10** | 7 | **13** | | 25 | **10** | 4 | **12** | 14 | | **9** | 3 | | **14** | 11 | | |  |
| Social context: | **1** | 2 | **2** | 8 | | **1** | 1 | **1** | | 1 | **2** | 1 | **3** | 3 | | **0** | 0 | | **4** | 3 | | |  |
| Death or disease of family member | 1 | 1 | 1 | 5 | | 0 | 0 | 0 | | 0 | 2 | 1 | 2 | 2 | | 0 | 0 | | 4 | 3 | | |  |
| Social vulnerability | 0 | 0 | 0 | 1 | | 0 | 0 | 0 | | 0 | 0 | 0 | 1 | 1 | | 0 | 0 | | 0 | 0 | | |  |
| Change of main healthcare professional | 1 | 1 | 0 | 1 | | 1 | 1 | 1 | | 1 | 0 | 0 | 0 | 0 | | 0 | 0 | | 0 | 0 | | |  |
| Opportunity initiation raised by other healthcare professional or family member: | **8** | 11 | **6** | 25 | | **10** | 7 | **6** | | 12 | **12** | 5 | **7** | 8 | | **6** | 2 | | **7** | 6 | | |  |

*as perceived optimal timing of ACP initiation is identified by up to 3 GPs per health record, the total number of identified triggers is high, compared to total number of triggers of actual timing.
